# There and Back Again: A Cytokine Receptor's Tail

**DOI:** 10.1097/HS9.0000000000000349

**Published:** 2020-02-20

**Authors:** David Kent

**Affiliations:** York Biomedical Research Institute, Department of Biology, University of York, York, YO10 5DD, United Kingdom.

In a membrane surrounding a blood cell, there lived a cytokine receptor. On either end of this membrane, a portion sticks out and faces either the inside or the outside of the cell and scientists have long debated how an extracellular signal (cytokine) translates into transcriptional changes. Newly published research in *Science*^1^ challenges decades of dogma by stating that cytokines facilitate receptor dimerization and goes on to suggest that the oncogenic function of JAK2 may actually be the result of catalyzing dimer formation from the cytoplasmic tail.

Decades of research in hematopoietic cell biology, and indeed entire laboratories, have focused on the structure and function of cytokine receptors since cytokine signaling is responsible for the majority of the decision-making in blood cell production (how much and what type of mature blood cell should be made). Cytokines are considered to be the “go” signals in hematopoiesis and dysregulation of cytokine signaling disturbs the homeostatic balance of blood cell production, leading to a number of diseases ranging from various cytopenias through to full blown leukemia.

In the hematopoietic system, household names such as EPO, TPO, GM-CSF, GCSF and the family of interleukins all bind Class I cytokine receptors that dimerise and signal through the JAK/STAT pathway. In the early 1990s, a landmark paper emerged with a fairly straightforward model of Class I cytokine receptor activation – a ligand binds and induces dimerization, thereby permitting JAK molecules to cross-phosphorylate.^2^ Since these early experiments though, prevailing wisdom has shifted to suggest that Class I cytokine receptors exist as pre-formed dimers.^3,4^ Initially, this left a substantial question: “How does a ligand that binds a pre-formed dimer on the outside of the cell actually induce the activation of JAK proteins on the inside of the cell?” Theories emerged based on crystal structures, epitope binding assays, and cell line work – making a fairly compelling case that the ligand did not induce receptor binding, but was rather involved in stimulating conformational changes in the receptor that would allow activation of the JAK molecules.^4,5^ These studies were nearly exclusively performed in non-living cells using techniques that would irreversibly cross-link dimers, such as protein fragment complementation or cysteine cross-linking.^6,7^ This amplified the chances of observing pre-formed dimers, but did not provide a sense of their relative abundance or their behavior in a living cell.

The new research from Wilmes/Hafer et al drives a substantial wedge in the middle of the pre-formed dimer theories by measuring cytokine receptor dynamics in living cells using dual-color single-molecule fluorescence imaging in combination with post-translational cell surface labeling. Studying EPOR, TPOR, and GHR, this study showed that receptors were predominantly monomeric and randomly distributed in the inactive state. When ligand was added, rapid dimerization ensued, which rather harks back to the initial Cunningham et al model of ligand-induced dimerization.

Of significant interest to the myeloproliferative neoplasm (MPN) community, JAK2 V617F mutated cells were also tested using these state-of-the-art live cell imaging techniques. They first observed that ligand-induced dimers were more common in the presence of JAK2 and remained stable in the absence of the JAK2 tyrosine kinase domain. Intriguingly, the removal of both the tyrosine kinase and the pseudokinase (PK) domain led to loss of dimer stability. This is of course the location of the JAK2 V617F mutation which is the most common mutation in the MPNs (>50% of patients bear this single point mutation) and suggests that the role of the mutation might be to induce dimerization in the absence of ligand (and therefore result in the inappropriate signalling that drives disease). Wilmes/Hafer et al then repeated their initial experiments in the absence of ligand with mutant versus non-mutant cells and demonstrated that the V617F mutation led to ligand-independent dimerization at 50% (TPOR), 25% (EPOR) and 10% (GHR) of the maximum levels of dimerization. This would appear to give support for a structural role of the PK domain and also gives a potential mechanism for how JAK2 V617F-mutant cells are hypersensitive to both EPO and TPO.

If it turns out that ligand-induced dimerization is the key to enacting a Class I cytokine receptor activation of JAK and diseases are driven by modulation of dimerization rates, this will have substantial implications for our understanding of both normal and malignant hematopoiesis. Molecules that modulate dimerization rates could be developed to counteract the pathogenic effects of ligand-independent signaling or could be used to stimulate the production of specific cell types outside the body for therapeutic purposes.

This is an exciting paper – it challenges a currently held dogma with a potentially disruptive technology. At the very least it will inspire efforts on both sides to work out the exact mechanism of JAK activation in relevant in vivo models and ideally in patient samples where aberrant JAK biology is pathogenic. Perhaps most importantly, it challenges researchers to understand the limitations of current experimental data and to continue to try and destabilize long-held hypotheses about “how things work”.
